# Efficacy of lyophilised bacteria-rich faecal sediment and supernatant with reduced bacterial count for treating patients with *Clostridioides difficile* Infection – A novel method for capsule faecal microbiota transfer

**DOI:** 10.3389/fcimb.2023.1041384

**Published:** 2023-01-23

**Authors:** Adorján Varga, Lilla Makszin, Anita Bufa, Dávid Sipos, Péter Kása, Szilárd Pál, Philip Rosenstiel, Felix Sommer, Béla Kocsis, Zoltán Péterfi

**Affiliations:** ^1^ 1^st^Department of Internal Medicine – Department of Infectology, University of Pécs, Medical School, Pécs, Hungary; ^2^ Department of Medical Microbiology and Immunology, University of Pécs, Medical School, Pécs, Hungary; ^3^ Institute of Bioanalysis, Medical School, and Szentágothai Research Center, University of Pécs, Pécs, Hungary; ^4^ Institute of Pharmaceutical Technology and Biopharmacy, University of Pécs, Faculty of Pharmacy, Pécs, Hungary; ^5^ Institute of Clinical Molecular Biology, Christian Albrechts University and University Hospital Schleswig-Holstein, Kiel, Germany

**Keywords:** FMT, Clostridioides difficile, capsule, lyophilisate, Clostridium difficile infection

## Abstract

**Background and aims:**

Faecal microbiota transfer (FMT) has managed to earn its place in the *Clostridioides difficile* infection (CDI) guidelines by having comparable efficacy and recurrence rate of fidaxomicin. After more than 100 successful FMT administration through nasogastric tube, we started using hard gelatine capsules filled with lyophilised faecal sediment and supernatant. Our main question was whether uncoated capsules (containing faecal sediment or supernatant) are comparable to the widely used nasogastric tubes in CDI. We also investigated the effect of storage and time on the survival rate of bacteria in the samples.

**Methods:**

We compared the efficacy of our capsules to other treatment options of CDI at the Department of Infectology at the University of Pécs (Hungary). For our study, stool was collected from a single donor. We treated 10 patients with relapsing CDI, 5 of them received supernatant, 5 received sediment. Donor samples were stored on 4 different temperatures and tested to determine the survival rates of bacteria. As pilot projects, we also assessed the changes of bacterial taxa, protein- and lipid compositions. Moreover, we selected 4 patients to compare their samples prior and after FMT by using microbiome (16S amplicon sequencing), protein, and lipid analyses.

**Results:**

4 out of the 5 patients who received supernatant became symptomless within 2 days after FMT. In the sediment group 3 out of 5 patients were cured from CDI. Comparing the supernatant to the sediment, we found significantly lower number of colony-forming units in the supernatant. We found that -80°C is the most suitable temperature to store the samples. The stool lipid profiles of recipients showed a more diverse composition after FMT, and changes in the stool protein profiles were observed as well. In the microbiome analysis, we observed an increase in the alpha diversity after FMT.

**Conclusions:**

Our study of 10 patients showed good efficacy of lyophilised faecal supernatant using capsules. The single donor approach proved to be effective in our investigation. A significantly lower CFU number was sufficient for the effect, the separation can be achieved by widely available instruments. For storage temperature, -20°C was sufficient in our clinical practice.

## Introduction

Being a major public health challenge, *Clostridioides difficile* infections brought a significant attention to nosocomial infections and to over-, or misuse of antibiotics. A study including 886 patients, aiming to determine risk factors for CDI showed that 86.8% were hospitalized and 82% of the patients were given antibiotics within a 3-month period prior to developing CDI (Vigvari et al., 2018). Restoration of the colonization resistance by antibiotics is an indirect method: suppressing the abundance of taxa that cause gastrointestinal symptoms and further use of antibiotics may lead to the restoration of symptoms, but in many cases it is not a viable option. The aim of FMT is to restore colonization resistance in the gut by introducing a healthy microbial community to the impaired ecosystem. Although several studies have proven the method efficient, we are still not sure about the mechanism behind the results. Recently, a phase 3, double-blind, randomized, placebo-controlled trial concluded a very good efficacy for using purified Firmicutes spores in capsules (Feuerstadt et al., 2022), other studies have already suggested beneficial effects of probiotics in the prevention of CDI (Na and Kelly, 2011). FMTs have already been acknowledged in international guidelines for the therapy of CDI. Fidaxomicin is known as the most effective antibiotic in CDI. A recent study found FMT superior to Fidaxomicin for treating CDI (Hvas et al., 2019). Further studies are needed to elucidate the feasibility of FMT in other conditions as inflammatory bowel diseases (Fang et al., 2018), diabetes (Harsch and Konturek, 2018), obesity (Harsch and Konturek, 2018) and psychiatric disorders (Kurokawa et al., 2018). FMT protocols share the strict donor screening criteria, but there are differences in donor selection. While some studies prefer close relatives, some have no preferences, and others suggest the use of pooled samples from multiple donors. Since we are yet unsure about the critical properties of the FMT samples, we do not know if there are universal donors (or universal acceptors). Some authors suggest the importance of the abundance of short chain fatty acid (SCFA) producing bacteria (Udayappan et al., 2014). If these species could be proven as crucial for the success of FMTs, then selection of suitable donors would be greatly facilitated. Previously, due to the lack of strong evidence, we were using donors, who were relatives to the patients, if possible. In some cases, however, it was not achievable as seen in a former study encompassing 30 patients. In that study 8 individuals received stool from non-related donors, 2 of them had recurrence, while in the other group receiving FMT from related donors only 1 patient experienced a following episode of CDI (Vigvari et al., 2015).

FMT is mostly administered in form of a faecal suspension. Some centers favour nasogastric (NG) or nasojejunal (NJ) tubes, while others use colonoscopy to administer the faecal suspension. NG administration seems to be more accessible as it does not require X-ray control as NJ and also does not require specialists or their equipment as colonoscopy, but all these routes have been shown equivalent with respect of efficacy (Garborg et al., 2010; Brandt et al., 2012; van Nood et al., 2013). A study of 60 patients showed that after NJ-FMT the symptoms resolved within a day and there were no recurrences while *via* NG tube 88.64% resolved within a day and a recurrence rate of 11.36% was observed (Vigvari et al., 2019a). In the same hospital, an earlier study with lower case number (15-15) showed comparable results. The primary cure rate was 100% in the NJ group, while being 80% for primary and 93.3% for secondary cure rate in the NG group (Vigvari et al., 2014). FMTs are mostly performed using fresh donor faecal material. However, being able to use stored material, e.g. frozen or lyophilised, would make FMT more accessible. In a study of 19 patients lyophilised inoculum was used. The samples were obtained from non-related donors and administered through NG tube. 15 patients became symptom-free after the first treatment, 2 patients needed a second FMT and 1 patient was cured with a subsequent antibiotic treatment (1 patient passed away due to unrelated diseases) (Vigvari et al., 2019b). The inconvenience of the mentioned routes of administration instigated clinicians to look for alternative administration routes. One plausible option was the use of capsules. Trials and studies have been published with capsules containing frozen stool (Youngster et al., 2016) and freeze-dried stool (Hecker et al., 2016; Varga et al., 2021). In our clinical practice we are using hard gelatine capsules filled with freeze-dried stool from a non-related donor. We decided to use one donor (not related to any patients) throughout the whole study, because the sample donations and the required laboratory test could be arranged much more efficiently. Furthermore, using the one donor approach eliminated a variable from our study. The method seem to perform similar to the formerly used NG tube with respect to efficacy, and patients find it much more acceptable. Our main goal was to test our workflow, if it can meet the needs of a clinical centre treating CDI patients. We also compared the efficacy of the bacteria rich sediment and the supernatant, which contains significantly less bacteria. As pilot projects for our future studies, we compared stool samples from CDI patients prior and after FMT. We also collected samples from healthy individuals. During these projects we carried out protein- and lipid composition analyses as well as microbiome analysis (16S amplicon sequencing) for 4 patients and for their FMT donor.

## Methods

### Study design

In our pilot study the enrolled patients received capsules filled with lyophilised faecal supernatant (*group 1*) or sediment (*group 2*). Sediment was defined as the portion of the sample that settled to the bottom of the centrifuge tube after 15 minutes with 3309 g and could be visibly distinguished from the opaque supernatant (Varga et al., 2021). The faecal transfers were carried out between January 2018 and January 2020. The application of FMT for the treatment of recurrent CDI has already been accepted by international guidelines. FMTs were carried out after the written informed consent of the patients.

We collected stool samples from 4 patients before FMT and after the cease of symptoms for lipid and protein profile and metagenome analysis.

Survival of the bacteria in the lyophilised samples was examined during storage in 4 different temperatures to determine the appropriate storage conditions and storage time.

### Study population

10 patients were included in our pilot study and divided into 2 groups. In *group 1*, 5 patients received capsules containing lyophilised faecal supernatant, whereas in *group 2*, 5 patients received capsules containing lyophilised faecal sediment (**Table 1**). The amount of the capsules depended on the physical characteristics of the actual lyophilised material. It varied between 4 and 6 capsules (hard gelatine, size ‘00’.) Patients admitted with recurrent CDI were tested for toxin A with *C. difficile* toxin enzyme-linked immunosorbent assay (Techlab *C. diff* Quik Chek Complete). If the test result confirmed a new episode of CDI, patients were informed about the possibility of FMT and included in the study. Patients were followed up for at least 6 months.

**Table 1:** Study population.

	Supernatant	Sediment	Combined
**Age**	71	68.8	69.9
**Sex (male/female)**	2/3	2/3	4/6
**Recurrence**	6.8	2.4	4.6
**Number of patients**	5	5	10

### Stool preparation

The donor was a 26 year-old Caucasian man, non-related to any of the patients. He had slim physique with normal BMI and was on a balanced diet. The donor was screened according to comprehensive guidelines (**Table 2**) (Wang et al., 2019; Gweon et al., 2022). Recent guidelines include testing for an extended spectrum of viruses associated for diarrhea (Rotavirus, Norovirus, Adenovirus, Astrovirus) using real time PCR, moreover, in pandemic periods COVID-19 tests (nasopharyngeal swab-antigen and PCR testing, serology for SARS-CoV-2, stool testing for SARS-CoV-2) have to be carried out (Gweon et al., 2022). Donor stool was collected at the donor’s home and stored in an airtight container during transfer to the laboratory. Stool was processed within 2 hours from collection under laminar airflow in a dedicated cabinet. 60 g of faeces was weighed and mixed with 200 mL of sterile physiological saline solution (0,9% sodium chloride). The mixing and homogenisation were carried out with a standard commercial handheld mixer (AEG HM 250). After 1 minute of homogenisation, the resulting slurry was filtered through a commercial colander and then through a commercial screen with smaller mesh size in order to remove larger debris. To achieve an even more homogenous solution, the sample was centrifuged for 10 minutes at 827 g (centrifuge model: MPW-380R, Poland). This step was necessary in order to remove smaller debris effectively, since even small debris make lyophilisation more complicated. The supernatant was divided into 100 mL portions, as this volume was used in our previous FMT protocol. The 100 mL portions were filled into 50 mL centrifugation tubes (Sarstedt, Nümbrecht, Germany) and centrifuged for 15 minutes at 3309 g. With this step we were able to separate the samples into a bacteria-rich sediment and a supernatant that contained significantly less bacteria. The supernatant and the sediment were frozen separately at -20°C and lyophilised (Freeze Dryer Heto Drywinner model DW1.0.) The freeze-drying was carried out under the following conditions: -40°C, 4*10^-4^ mbar, 36 hours. Following lyophilisation, samples were homogenised in a mortar with a pestle and filled into an appropriate number (4-6) of ‘00’ sized hard gelatine capsules with a commercial capsule filling tool (Capsule Machine, Capsule Connection, LLC, Prescott AR. USA.) The capsules were stored at -20°C until administration.

**Table 2:** Suggested laboratory tests for potential donors for faecal microbiota transplantation.

Tests	Blood	Stool	Others
**Bacteria**	*Treponema* spp.	Enteric pathogen culture: *Salmonella, Shigella, Campylobacter* spp. *Helicobacter pylori –* EIA VRE antibiotic sensitivity test to prevent the use of stool containing polyresistant strains.	
**Viruses**	Hepatitis A virus IgM Hepatitis B virus surface antigen Anti-hepatitis C virus antibody HIV 1 and 2 Serology for SARS-CoV-2	Norovirus EIA or PCR Rotavirus EIA or PCR Adenovirus PCR Astrovirus PCR SARS-CoV-2	COVID-19 tests (nasopharyngeal swab - antigen and PCR testing)
**Parasites**	*Entamoeba histolytica* *Strongyloides stercoralis*	Ovum and parasite *Microsporidia* *Giardia* faecal antigen/EIA *Cryptosporidium* EIA AFB for *Isospora* and *Cyclospora*	
**Others**	Complete blood count Liver function test ESR and CRP	*Clostridioides difficile* test PCR of toxin genes	

AFB – Acid Fast Bacillus; CRP – C Reactive Protein; EIA – Enzyme Immuno Assay; ESR – Erythrocyte Sedimentation Rate; HIV – Human Immunodeficiency Virus; IgM – Immunoglobulin M; PCR – Polymerase Chain Reaction; SARS-CoV-2 – Severe Acute Respiratory Syndrome Coronavirus 2; COVID-19 – Coronavirus Disease 2019.

### Administration of the capsules

Patients were instructed to avoid eating on the day of the FMT. All patients received vancomycin or metronidazole prior to FMT. Prokinetic and proton pump inhibitor were given as premedication. After the procedure, patients were told to stay in a 45° upright position for 2 hours. Patients were discharged within 2 days if they became symptom-free and if no adverse events occurred.

### Patient follow-up

Patient follow-up was scheduled for 1, 3 and 6 months after FMT.

### Sample preparation for microbiota analysis

Stool samples were mixed with sterile physiological saline solution (0.9% sodium chloride) and filtered through disposable paper wool. 1.5 mL was filled into Eppendorf tubes for storage under -80°C until the analyses were carried out. Samples for metagenome analysis were filled into OMNIgene OMR-200 transport tubes (DNA Genotek, Canada) according to the instructions of the manufacturer.

### Microbiota analysis using 16S amplicon sequencing of donor and patient samples

DNA was isolated from faecal material using the DNeasy PowerSoil Kit (Qiagen) following the manufacturer’s protocol. Extracted DNA was eluted from the spin filter silica membrane with 100 µl of elution buffer and stored at -80°C. 16S profiling and MiSeq sequencing was performed as described earlier (Sommer et al., 2014; Sommer et al., 2016) with the following modifications: the V3-V4 region of the 16S gene was amplified using the dual barcoded primers 341F (GTGCCAGCMGCCGCGGTAA) and 806R (GGACTACHVGGGTWTCTAAT). Each primer contained additional sequences for a 12 base Golay barcode, Illumina adaptor and a linker sequence (Caporaso et al., 2012).

PCR was performed using the Phusion Hot Start Flex 2X Master Mix (NEB) in a GeneAmp PCR system 9700 (Applied Biosystems) and the following program (98°C for 3 min, 25x (98°C for 20 s, 55°C for 30 s, 72°C for 45 s), 72°C for 10 min, hold at 4°C). Performance of the PCR reactions was checked using agarose gel electrophoresis. Normalization was performed using the SequalPrep Normalization Plate Kit (Thermo Fisher Scientific, Darmstadt, Germany) following the manufacturer’s instructions. Equal volumes of SequalPrep-normalized amplicons were pooled and sequenced on an Illumina MiSeq (2 x 300 nt). MiSeq sequence data was analyzed using MacQIIME v1.9.1 ( , ). Briefly, all sequencing reads were trimmed keeping only nucleotides with a Phred quality score of at least 20, then paired-end assembled and mapped onto the different samples using the barcode information. Rarefaction was performed at 4062 reads per sample to normalize all samples to the minimum shared read count and to account for differential sequencing depth. Sequences were assigned to operational taxonomic units (OTUs) using uclust and the greengenes reference database (gg_13_8 release) with 97% identity. Representative OTUs were picked and taxonomy assigned using uclust and the greengenes database. Quality filtering was performed by removing chimeric sequences using ChimeraSlayer and by removing singletons and sequences that failed to align with PyNAST. The reference phylogenetic tree was constructed using FastTree 2. Relative abundance was calculated by dividing the number of reads for an OTU by the total number of sequences in the sample. Beta diversity was calculated using Bray Curtis dissimilarity and visualized by principal coordinate plots. Alpha diversity was assessed by comparing the total number of OTUs, Chao1 richness, Shannon entropy and Simpson’s index. Differentially abundant taxa were assessed using nonparametric t test and p values were adjusted for multiple testing using FDR correction.

### Short chain fatty acid analysis

We examined short chain fatty acids between C2:0 and C7:0. We were especially interested in the differences and changes of butyrate (C4:0) levels of the samples.

Stool samples from patients and healthy individuals were stored in small containers in -80°C. The patient samples were collected before and after FMT. Furthermore, native donor samples were compared to lyophilised samples.

Due to major differences between the concentrations of the samples, a qualitative analysis of the SCFA levels was carried out. 100 mg of the sample was diluted in 1 mL distilled water, followed by 10 minutes of vortexing. The samples were then incubated in room temperature for 10 minutes, then centrifuged with 10000 RPM (15000 g) for 10 minutes. The supernatant was collected, and mixed with the internal standard solution. The samples were then filtered (Low Protein Binding Hydrophilic LCR (PTFE) Membrane 0.2µm, Millex^®^, Tullagreen, Ireland) into GC tubes for the gas chromatography.

An Agilent Technologies 6890N gas chromatograph with a 5975 mass selective detector (Agilent, Waldbronn, Germany) was used for the analysis. Data analysis was performed using the GC/MSD CHEMSTATION (Version D.03.01, Agilent) software. Components were identified with the help of the NIST MS Search 2.0 library ( , ) and by spiking the samples with standards. C7:0 in its methyl ester form was used as an internal standard.

### Faecal protein profile analysis

The collected stool samples were analysed with microchip gel electrophoresis (protocol below from Makszin et al. (2018)). As mentioned above, there were major differences between the concentrations of the samples, therefore a qualitative analysis of the protein levels was carried out.

We also analysed the faecal protein composition of donor samples prior and after lyophilisation. In this experiment, we aimed to access the effect of lyophilisation and storage time of lyophilised samples on the protein composition.

Electrophoresis in microchips was performed with the High Sensitivity Protein 250 LabChip kit in the commercially available Agilent 2100 Bioanalyzer system (Agilent Technologies, Waldbronn, Germany) equipped with a diode laser for fluorescence detection with 630 nm excitation and 680 nm emission wavelengths, as described previously (Makszin et al., 2012). The kit included microchips and reagents, such as High Sensitivity Protein 250 Labeling Dye, DMSO, ethanolamine, Protein 250 Standard Labeling Buffer (SLB, 300 mM Tris/HCl, pH >8.5), Gel Matrix (4.5% polydimethyl acrylamide - based linear polymer solution at pH 8), Destaining Solution, and Sample Buffer. The denaturing solution containing SDS and dithiothreitol (DTT) was prepared by adding 3.5 µL 1 M DTT (Boehringer Mannheim GmbH, Mannheim, Germany) to 100 µL Sample Buffer. The protocol was optimized for human stool proteins to achieve good separation and sensitivity.

The sample preparation consisted of a centrifugation with 3500 RPM (1500 g) for 5 minutes, separating of 4,5 µL supernatant, and adding 0.5µL SLB solution. The fluorescently labelled proteins were prepared by mixing 4.5 µL sample volume with 0.5 µL diluted fluorescent dye/DMSO solution and incubated for 10 min at room temperature. The excess dye (i.e. the unbound dye) was quenched by adding 0.5 µL ethanolamine and incubated for 10 minutes at room temperature. The labelled samples were diluted two times by adding 6 µL deionized water. 4 µL of this diluted sample solutions were combined with 2 µL denaturing solution, incubated at 100°C for 5 min, and centrifuged. 6 µL of each sample were loaded on the microchip channels filled with a polydimethyl-acrylamide-based linear polymer solution (pH 8). The respective well was loaded with the Destaining Solution.

Samples were injected with 1000 V for 80 s (injection volume was ca. 40 pL), and the separation was continued toward the anode at 1000 V for 60 s at 30°C. Each sample was analysed at least three times. The molecular masses of the protein components were determined by using the calibration curves in ref (Makszin et al., 2012). From the area under the curve (AUC) of the components, relative proportions were calculated and expressed as % of total AUC.

### Survival of bacteria during storage

Altogether, we received 9 stool samples from our donor. At the end of the lyophilisation, portions of 10 mg were filled into Eppendorf tubes for storage. Colony forming unit (CFU) number was counted at 6 different time points: end of lyophilisation, 2 days, 2 weeks, 1 month, 3 months and 6 months after lyophilisation. Samples were stored at 4 different temperatures: +20°C, +4°C, -20°C and -80°C. Samples were re-suspended to their initial concentration and diluted to enable CFU analysis *via* counting of colonies on agar plates. The following media were used: blood agar for aerobic and anaerobic culturing, eosin methylene blue agar, chocolate blood agar, chocolate blood agar with vancomycin, Sabouraud agar for aerobic culturing. CFU number was counted after 48 hours.

## Results

### Patient characteristics

10 patients were included in the study, they were randomized into groups. Both groups consisted of 5 patients. The individuals in the two groups were comparable in respect of age, sex, number of recurrences (except for *Patient #5*, who had 22 recurrences). They also received similar treatment for the CDI prior to FMT. Samples from 4 patients (*Patient #1*, *#2*, *#6* and *#9*) were sent for microbiome, protein and lipid level analysis (**Tables 3A, B**).

**Table 3:** Basic characteristics of FMT patients – supernatant (a) and sediment (b) group.

Oszlop1	Patient #1	Patient #2	Patient #3	Patient #4	Patient #5
**Group**	Supernatant	Supernatant	Supernatant	Supernatant	Supernatant
**Age**	83	82	66	62	62
**Sex**	Male	Female	Female	Female	Male
**Number of recurrences**	4	2	4	2	22
**Previous antibiotics**	metronidazole, vancomycin, fidaxomicin	metronidazole, vancomycin, fidaxomicin	metronidazole, vancomycin, fidaxomicin	metronidazole	metronidazole, vancomycin, fidaxomicin
**Risk factors**	rCDI	radiation colitis, small bowel resection, UTI - augmentin (amoxicillin + clavulanic acid), ceftriaxon, hospitalisation, rCDI	IBD, colectomy rCDI	rCDI	rCDI
**Firs FMT successful?**	Yes	Yes	Yes	Yes	No
b					
**Oszlop1**	**Patient #6**	**Patient #7**	**Patient #8**	**Patient #9**	**Patient #10**
**Group**	Sediment	Sediment	Sediment	Sediment	Sediment
**Age**	82	43	76	66	77
**Sex**	Female	Male	Female	Male	Female
**Number of recurrences**	2	2	3	3	2
**Previous antibiotics**	metronidazole, vancomycin	metronidazole, vancomycin	metronidazole, vancomycin, fidaxomicin	vancomycin	metronidazole, vancomycin
**Risk factors**	UTI - ceftriaxon, rCDI	Antibiotic treatment against respiratory tract infection, rCDI	UTI - levofloxacin, pneumonia - imipenem, ceftriaxon, rCDI	augmentin (amoxicillin + clavulanic acid)	imipenem, cilastatin, augmentin (amoxicillin + clavulanic acid), rifampicin

### Baseline characteristics of patients

No patients dropped out during the study and no patients were excluded from the analysis.

The primary end point showed an overall 80% success rate; 90,9% in group 1 and 50% in group 2.

### Case histories and outcomes

*Patient 1* was an 83-year-old Caucasian man with type 2 diabetes mellitus and hypertension, hospitalized because of his fourth recurrence of CDI over a 3 month period (CDI was confirmed by testing for *C. difficile* toxin A). His infection was tried to treat with metronidazole, vancomycin and fidaxomicin without success. After obtaining faecal sample for further analysis, the patient received capsules containing lyophilised faecal supernatant (like all the other patients in group 1) in outpatient care. The symptoms stopped within 2 days after the procedure, a second faecal sample was collected in order to compare the pre- and post FMT and the donor samples.

*Patient 2* was an 82 years old Caucasian woman with type 2 diabetes mellitus and hypertension, presented with 2 recurrences of CDI within 2 months. Her first episode of CDI occurred during a *Klebsiella pneumoniae* (ESBL producing) sepsis which was treated by imipenem and cilastatin. The third recurrence was not responsive for vancomycin. Following stool sample collection, the patient received capsules containing lyophilised faecal supernatant (group 1.) After FMT, she passed type 4 stool 2 times a day (according to Bristol Stool Chart), she showed no side effects and was discharged after obtaining a second faecal sample. The patient remained symptom-free until the end of the follow up (2 years.)

*Patient 6*, an 82 years old Caucasian woman with type 2 diabetes mellitus, hypertension and rheumatologic disorders, was admitted after 2 episodes of CDI. The first episode of her CDI was treated with metronidazole, but after a temporary symptomless period, a recurrence occurred, which could not be cured with vancomycin. She belonged to group 2, therefore she received capsules filled with lyophilised faecal sediment. The patient became symptom-free after 3 days. She has not experienced further recurrences during the follow up. Faecal samples were collected prior and after FMT.

*Patient 9* was a 66 years old Caucasian man with positive alcohol history, who was admitted from the ER unit with elevated inflammatory markers and acute kidney failure. His diarrhea started during amoxicillin/clavulanic acid treatment. Vancomycin seemed to be effective for a short period of time, but his diarrhea started again soon. Vancomycin was not effective anymore, therefore FMT was planned. Belonging to group 2, he received capsules filled with lyophilised faecal sediment. Since the patient did not become symptom-free, a second FMT was carried out from the same donor sample as before. Following the second FMT he became symptom-free. Faecal samples were collected before the first dose of capsules, and after the successful FMT.

### Faecal bacterial profile analysis in patients and in the donor

Stool samples from the four randomly selected patients underwent 16 S ribosomal RNA gene-based microbiota analysis. The post-FMT sample from *Patient #9* did not yield sufficient sequencing reads and thus was excluded from the analysis.

**Figure 1:**
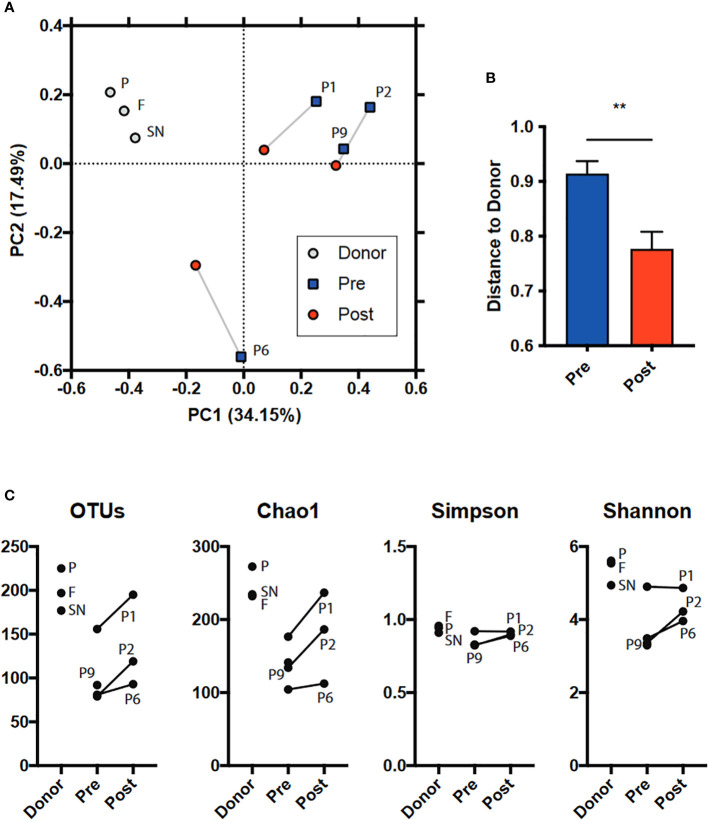
Microbiome diversity analysis including **(A, B)** beta (between samples, PCoA based on BrayCurtis dissimilarity and BC distance of “pre” and “post” FMT microbiomes to donor) and **(C)** alpha (within sample, several measures: number of observed OTUs; Chao1, Shannon, Simpson indices). SN: Supernatant. P: Pellet. F: Native faeces. Regarding how the lyophilisates differ from the fresh faeces, the taxonomy overview shows changes in the relative abundances of bacterial taxa during sample preparation and/or lyophilisation (**Figure 2**).

Due to the high interindividual variation and the low sample size, we could not detect any statistically significant taxonomic differences. However, in the Principal Coordinate Analysis (PCoA) plot (**Figure 1A**) the “after” samples move towards the donor. The distance to the donor significantly decreased indicating that the post-FMT samples became more similar to the healthy donor microbiome (**Figure 1B**). Similarly, in the alpha diversity measures, the “post” samples tend towards the donor by having and increased diversity (**Figure 1C**).

**Figure 2:**
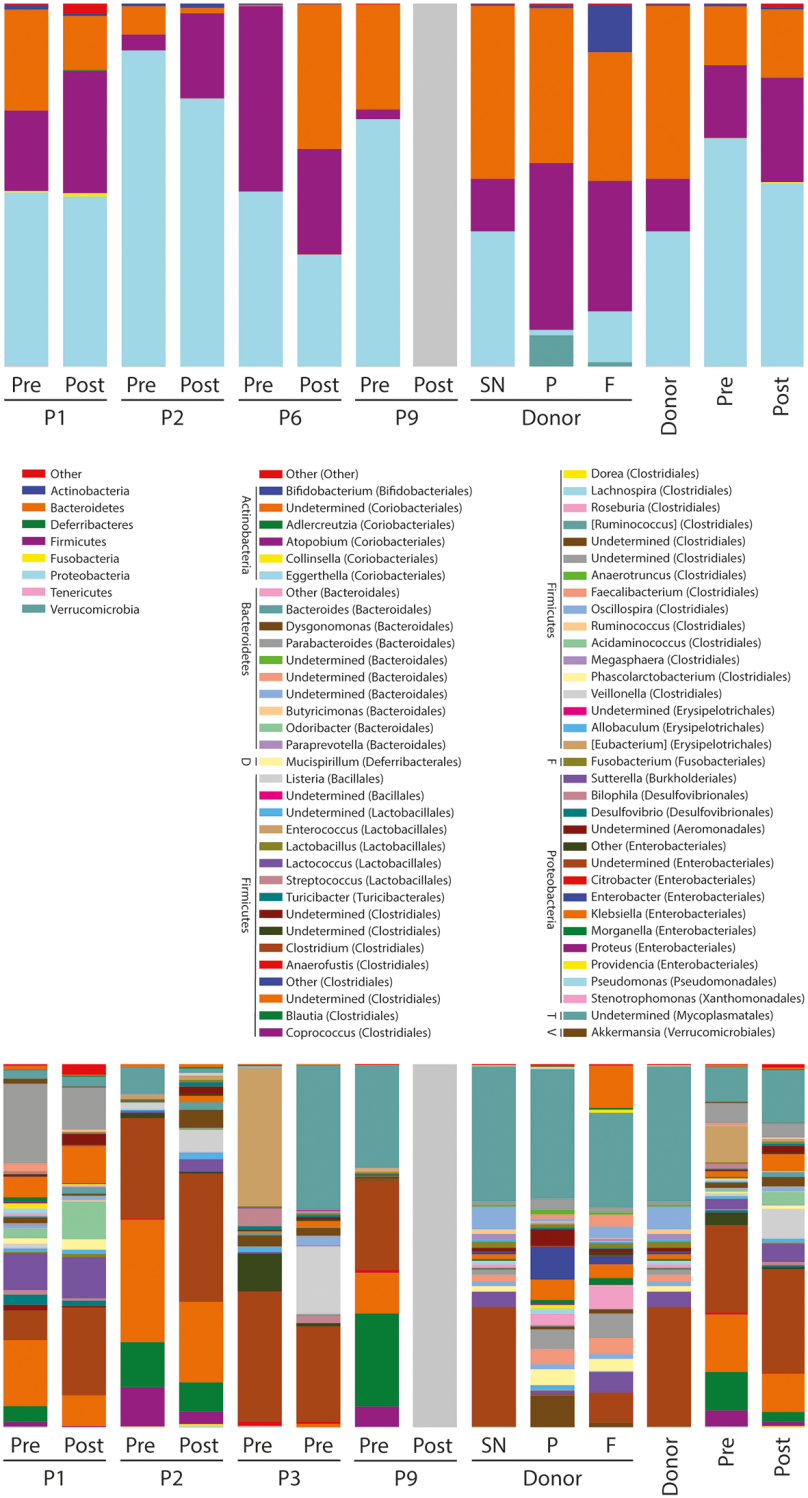
Taxa summaries on phylum (top) and genus (bottom) level. The bars on the right are means based on the sample groupings. Pre: the mean of the four pre-FMT samples. Post: the mean of three post-FMT samples. SN: Supernatant. P: Pellet. F: Native faeces. Phylum abbreviations in the genera legend: D: Deferribacteres. F: Fusobacteria. T: Tenericutes. V: Verrucomicrobia.

### Short chain fatty acid analysis

**Figure 3:**
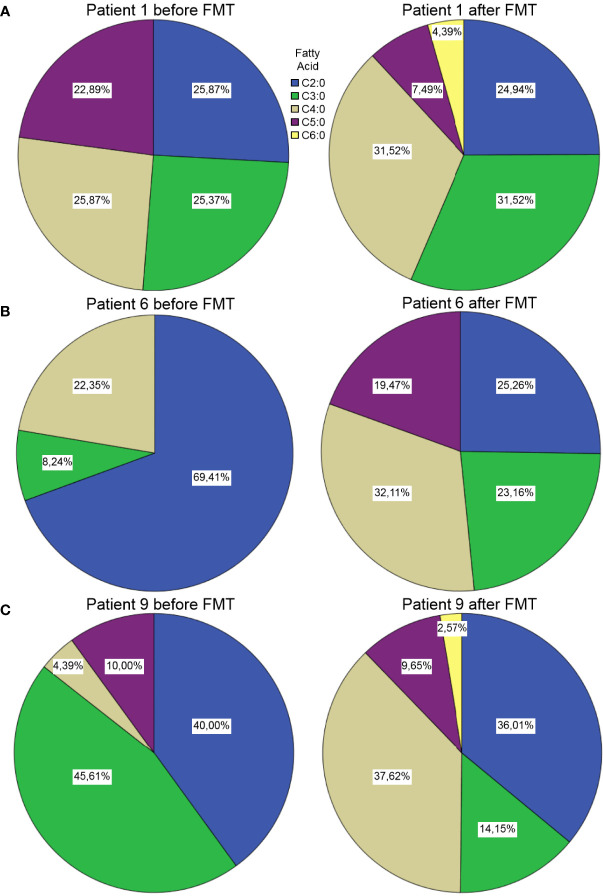
Short chain fatty acid compositions in samples from *Patient #1* **(A)**, *Patient #6* **(B)** and *Patient #9* **(C)** before and after FMT. Unfortunately, the pre-FMT stool sample from *Patient #2* (**Figure 4**) was not sufficient for this analysis (we did not see any spikes in the GC-MS results).

We observed differences on multiple levels among the samples. First of all, 5 out of the 6 healthy samples showed a more diverse composition of SCFA’s, than the pre-FMT samples. We also observed a shift towards a more diverse composition of SCFA’s after FMT in the samples obtained from the CDI patients. We found a prominent increase in the ratio of the C4:0 component after FMT. In the samples of *Patient #1*, the ratios of C4:0 and C5:0 increased, whereas C3:0 and C6:0 decreased (**Figure 3A**).

The sample of *Patient #6* showed a dominance of C2:0 prior to FMT, whereas the post-FMT

sample showed a rather divers composition of short chain fatty acids (**Figure 3B**).

In the case of *Patient #9*, the SCFA analysis showed an increase of the ratio of butyrates (C4:0), while the ratio of the propionates (C3:0) decreased after FMT (**Figure 3C**).

**Figure 4:**
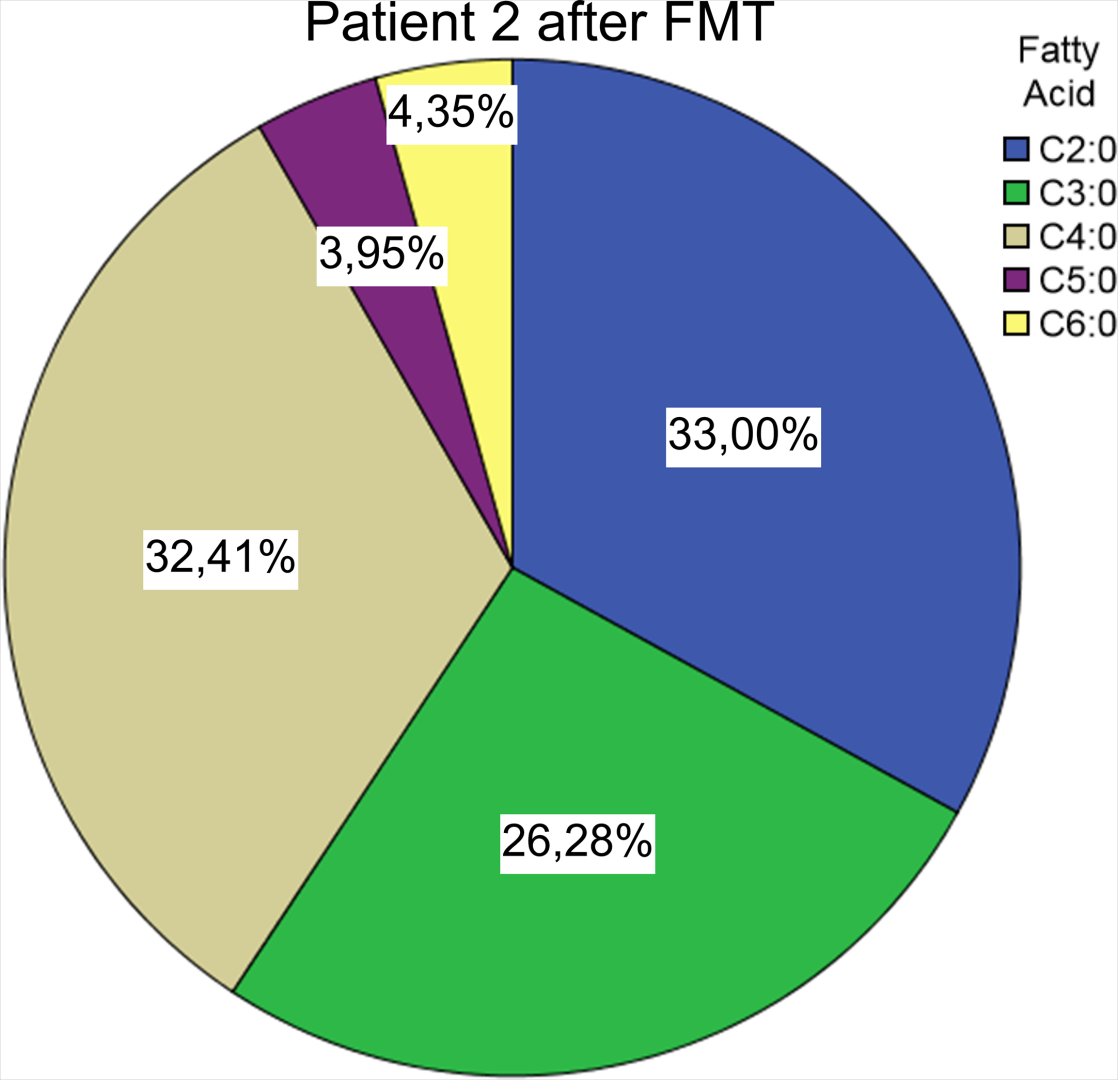
Short chain fatty acid compositions after FMT from *Patient #2* We also found the C4:0 component to be dominant in 5 of the 6 healthy donor samples, 5 out of 6 samples showed a rather diverse composition of SCFAs than the patient’s pre-FMT samples (**Figure 5**).

**Figure 5:**
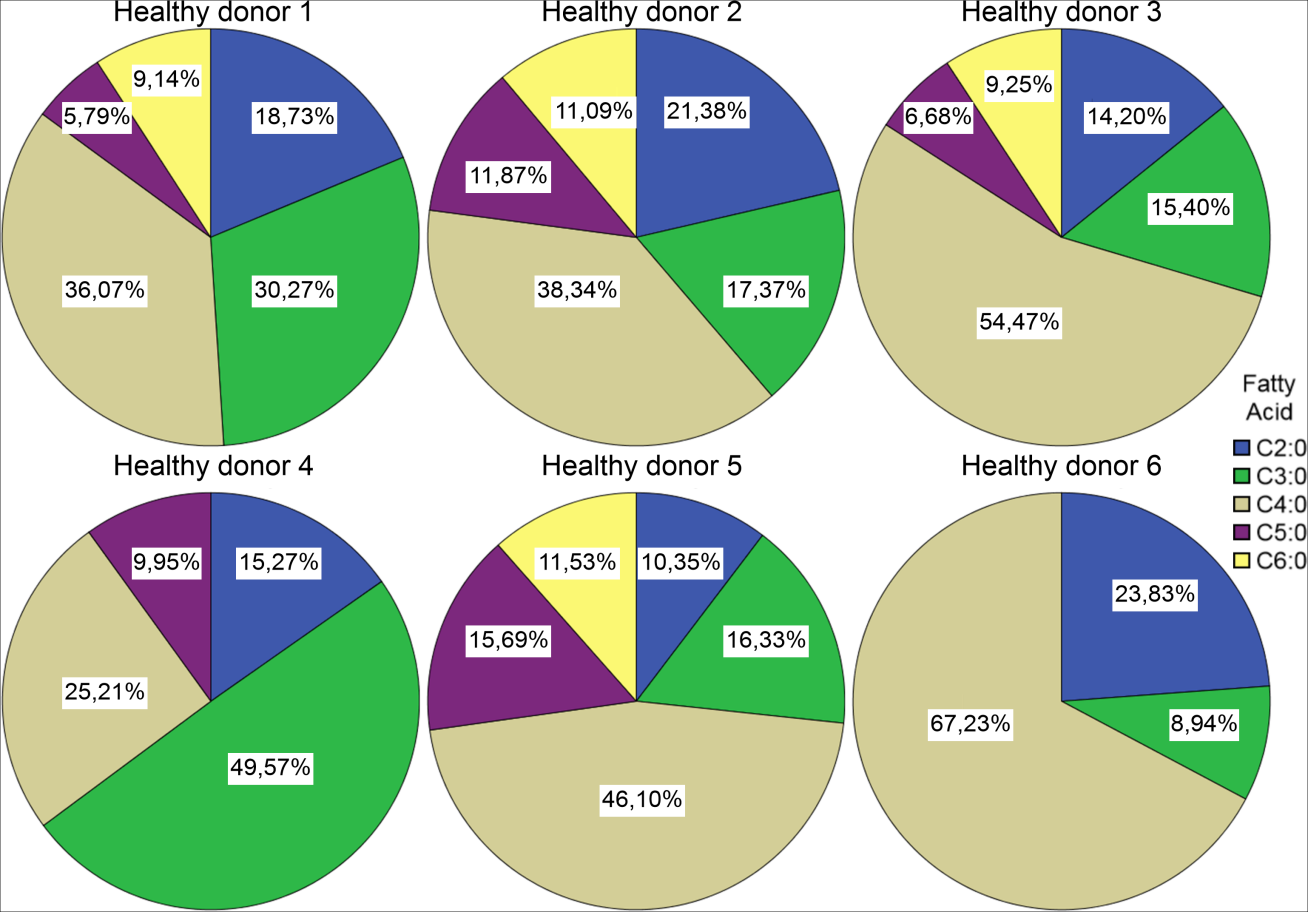
Short chain fatty acid compositions in samples from healthy donors. Comparing the compositions of pre- and post-lyophilisation samples of the FMT donor, it seems that the lyophilisation altered the relative abundances of the SCFAs: the ratio of the C4:0 component decreased, while the ratio of C2:0 increased (**Figure 6**).

**Figure 6:**
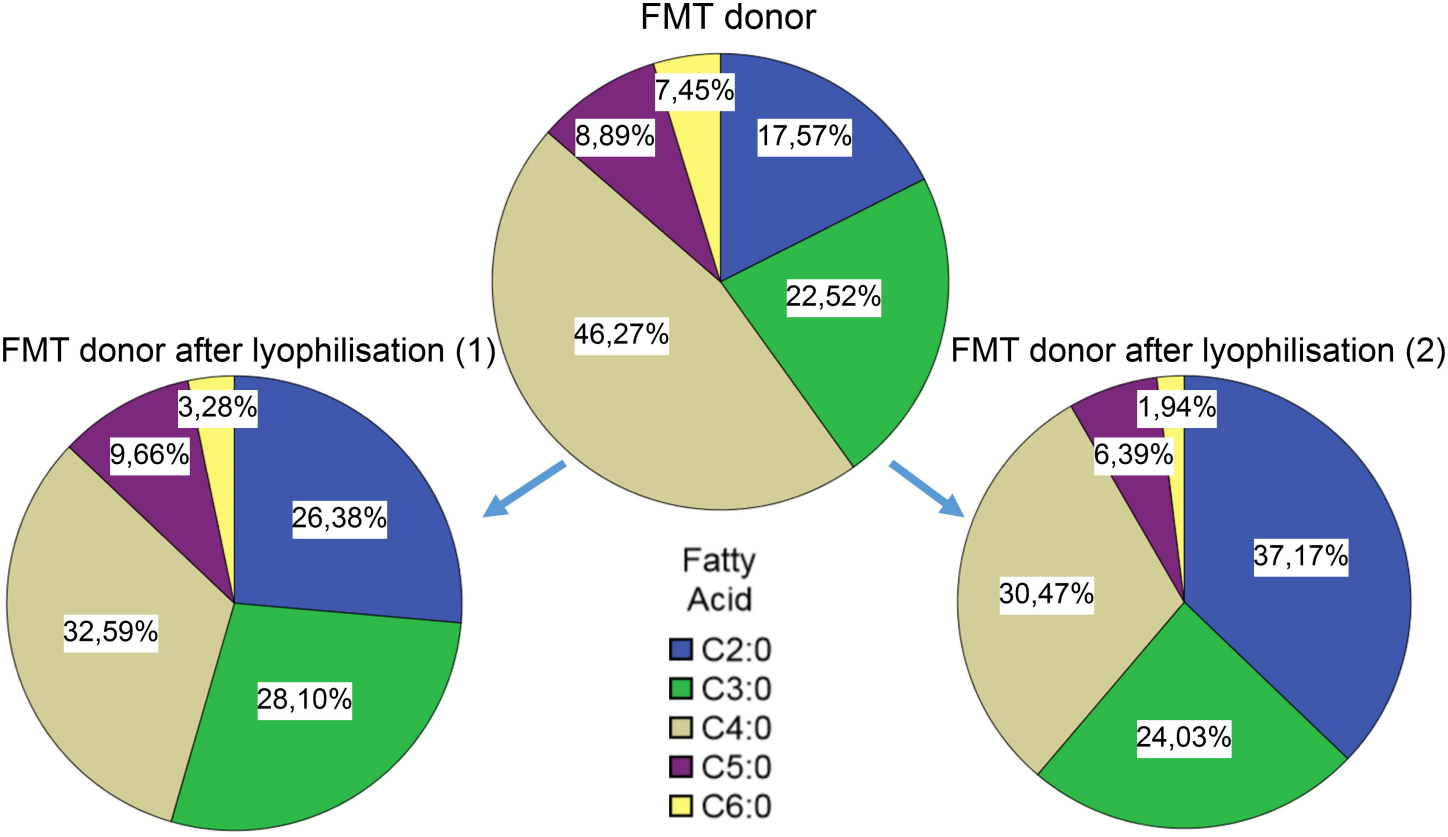
Short chain fatty acid compositions in donor samples before and after lyophilisation.

### Faecal protein profile analysis

Similar to the SCFA profiling, we faced difficulties due to the characteristics of the patient samples. We did not manage to obtain useful data from *Patient #1*’s sample.

**Figure 7:**
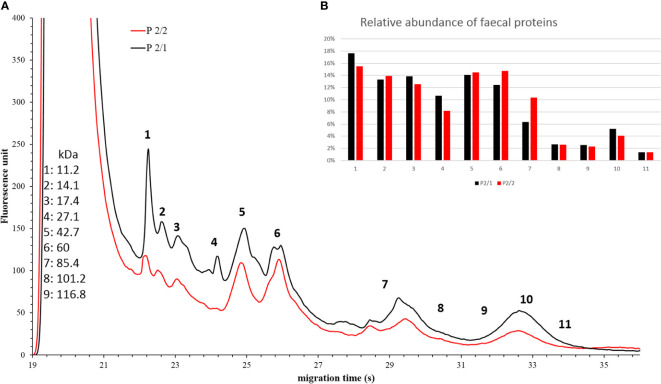
Faecal protein analysis **(A)** and relative abundance of faecal proteins **(B)** of *Patient #2* before (black) and after (red) FMT. The numbered data points refer to different components. The molecular weights of the components can be seen on the left side in kilodaltons. Panel b shows the relative abundance of these components. In the post-FMT samples from *Patients #6*, we observed an increase in the relative abundance of component 4, 5, 6 and 7 (**Figure 8**).

In the case of *Patient #2*, the relative abundances of component 7 increased (**Figure 7**).

**Figure 8:**
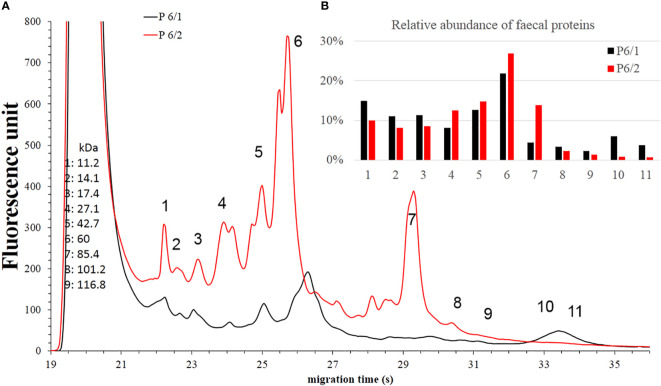
Faecal protein analysis **(A)** and relative abundance of faecal proteins **(B)** of *Patient #6* before (black) and after (red) FMT. The numbered data points refer to different components. The molecular weights of the components can be seen on the left side in kilodaltons. Panel b shows the relative abundance of these components. In the samples of *Patient #9*, the AUC% value only increased in the case of component 5 (**Figure 9**).

**Figure 9:**
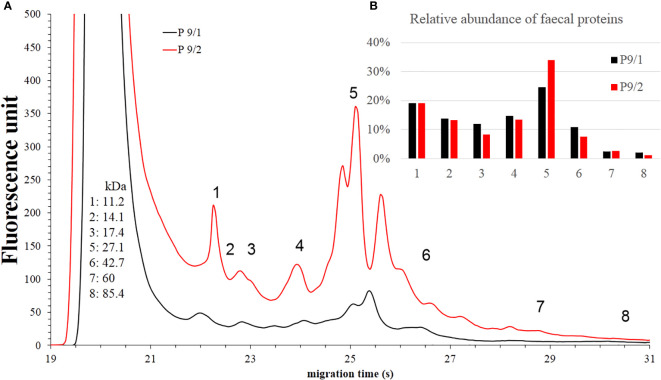
Faecal protein analysis **(A)** and relative abundance of faecal proteins **(B)** of *Patient #9* before (black) and after (red) FMT. The numbered data points refer to different components. The molecular weights of the components can be seen on the left side in kilodaltons. Panel b shows the relative abundance of these components.

### Donor samples

**Figure 10:**
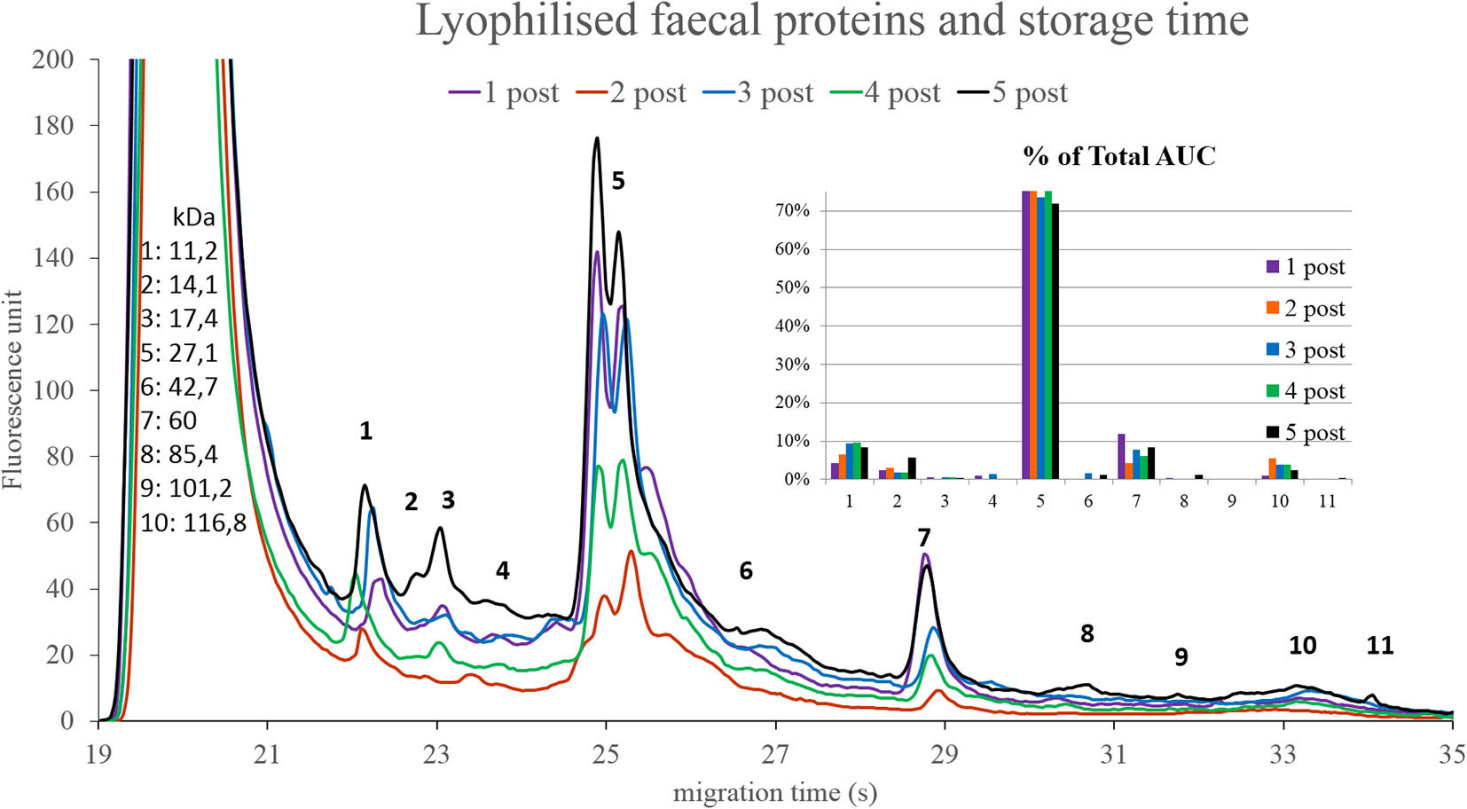
Protein compositions of lyophilised donor stool samples over time. The numbered data points refer to different components. The molecular weights of the components can be seen on the left side in kilodaltons. The bar graph on the right side shows the relative abundance of these components in the five samples. 1 post: 2 days, 2 post: 2 weeks, 3 post: 1 month, 4 post: 3 months 5 post: 6 months.

**Figure 11:**
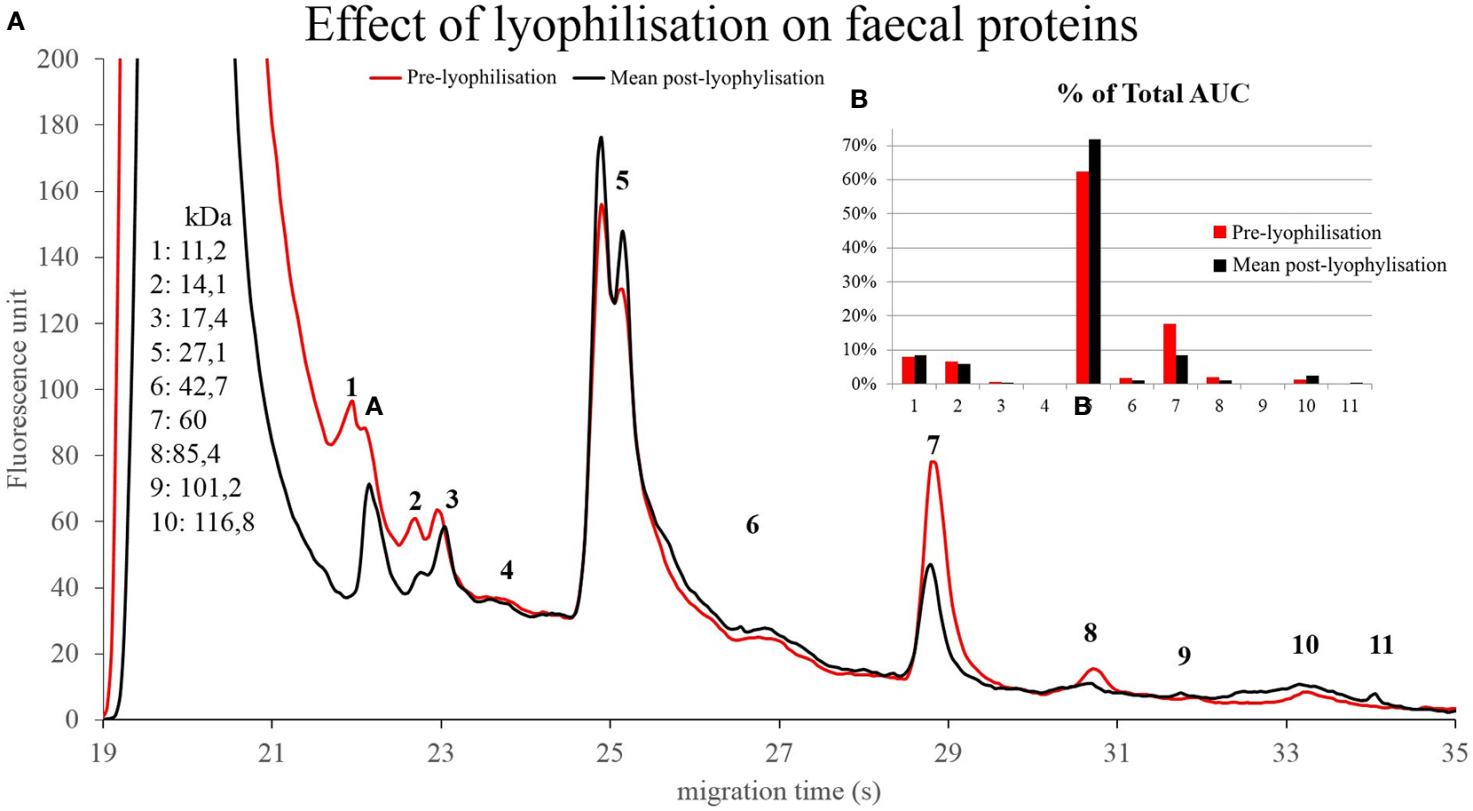
Protein compositions of donor stool before (red) and after (black) lyophilisation **(A)**. The numbered data points refer to different components. The molecular weights of the components can be seen on the left side in kilodaltons. The bar graph on the right side **(B)** compares the relative abundance of the components in the pre-lyophilisation and the post-lyophilisation samples.

Changes can be observed mainly in the AUC% of components 5 and 7. It seems, that both storage time (**Figure 10**) and lyophilisation (**Figure 11**) affects the protein composition of the faecal samples. Component 5 showed changes in the case of the pre- and post-FMT samples as well, although it is unclear which proteins are contained in that peak, and which significance these components have.

### Survival rate of the bacteria

**Figure 12:**
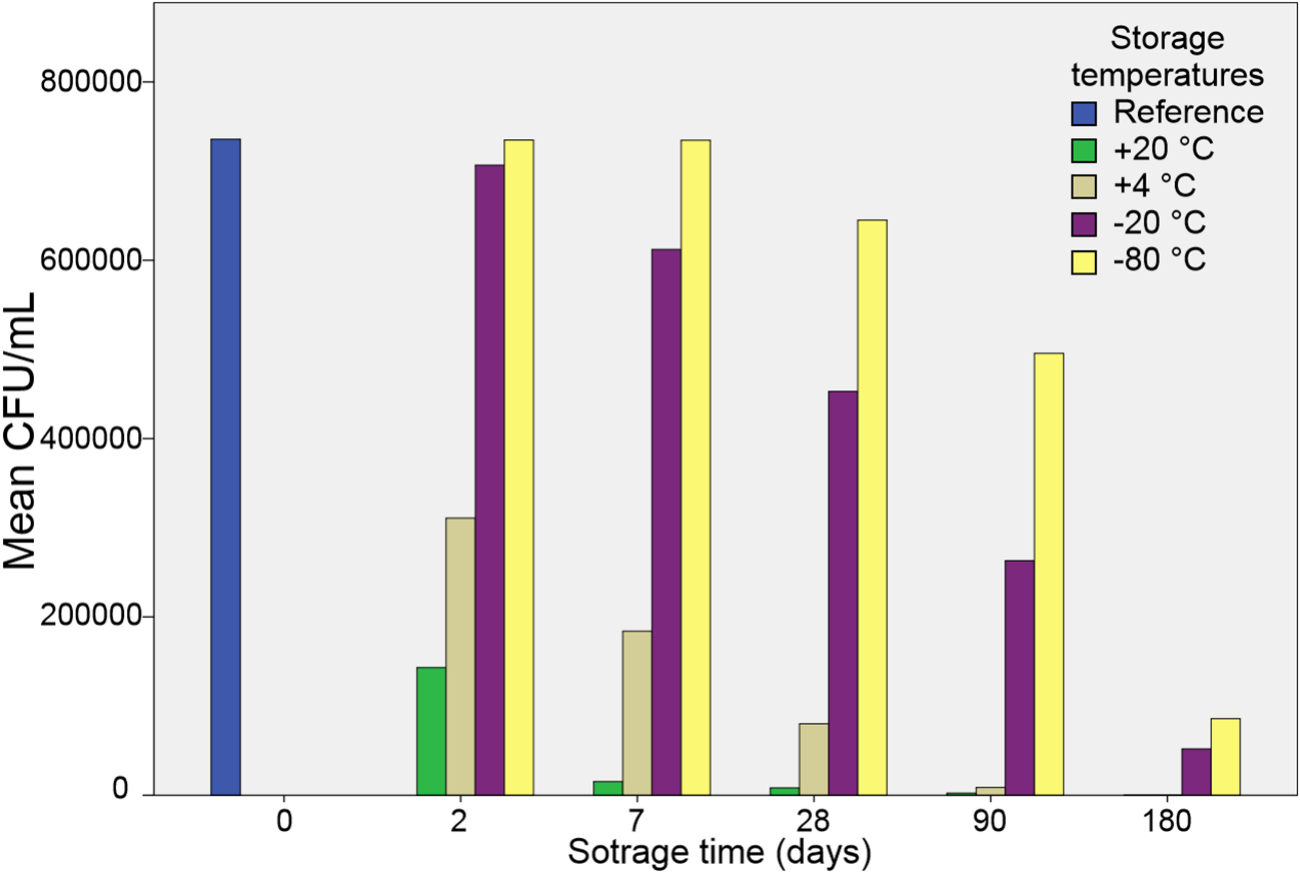
Colony forming units over storage time and storage temperature in sediment samples. The bars represent means of CFU’s counted on different aerobic and anaerobic agar plates.

Our experiments showed clearly, that a storage temperature of -80°C has the best results in the terms of survival rate. The survival rates decrease over time, and higher temperatures have worse results as well (**Figure 12**). We have to note though, that for our clinical experiment we only used stool samples stored on -20°C, and this temperature was sufficient to maintain the effectiveness of the lyophilised samples for at least 6 months.

The blue bar (“Reference”) on **Figure 12** represents the CFU/mL count in the sediment of fresh faecal sample. We did not assess the survival rates in supernatant samples, since there was an initial 600-fold difference in favour of the sediment. Moreover, we noticed in the early stage of the study that CFU numbers in stored supernatant samples dropped to 0 or became inconsistent after 2 months. The aerobic and anaerobic survival showed a similar pattern, although the CFU’s on the anaerobic culture media were much more abundant (this has been published in our previous article (Varga et al., 2021).

## Discussion

Our study proves the feasibility of an easy to replicate method of using lyophilised encapsulated stool in CDI. It has to be noted, that the tools required for the process are simple and widely available. As FMT has already earned its place in the international guidelines for CDI, every change to the method has to achieve the success rate observed with conventional FMT. By making the process less invasive (thus more tolerable for patients) and more flexible, our approach opens up the possibility for higher numbers of FMTs in CDI, furthermore it makes it a more feasible option in other potential fields of use. As we demonstrated here, capsules containing faecal supernatant (with significantly lower CFUs) may potentially serve as an alternative to the standard process, which supports the presumptions regarding the higher role of non-bacterial elements in the mechanism in the background (Ott et al., 2017).

The SCFA analyses imply a shift of SCFA profiles after FMT towards or more diverse composition. Publications showed the importance of SCFA’s in the healthy gut microbiome, especially the importance of butyrate (den Besten et al., 2013; Aden et al., 2019; Martin-Gallausiaux et al., 2020; Hinrichsen et al., 2021; Effenberger et al., 2021). This can be either a cause (as it provides a supportive environment), or an effect of a healthy microbiom (high abundance of butyrate producing bacteria). Our experiment to compare SCFA profiles of healthy individuals and CDI patients showed a more diverse SCFA composition, and higher relative ratios of butyrate’s in the healthy group as well. We have also observed changes in the SCFA profile upon lyophilisation – this could be due to different sublimation dynamics of the different fatty acids.

At this point it is difficult to conclude the results of our protein profiling experiments. On the other hand, it is worth mentioning, that changes can be observed in the protein profiles of CDI patients after FMT. Further trials are needed to compare healthy samples with pre- and post-FMT samples from CDI patients, including the identification of the proteins in component 5 and 7 on **Figure 10**.

Analysing stool proteins brings many additional problems. According to a metaproteomics study an optimised LC-MS/MS workflow of mouse faeces resulted in the identification of 18000 non-redundant tryptic peptides, 93% of these peptides were of microbial origin. They represented over 600 microbial species and 250 protein families (Lai et al., 2019). Another option is the analysis of mucosal lavage. A study showed a mixture of human proteins (up to 63%) and bacterial peptides (30%) (Li et al., 2011). By comparison, after analysing protein isolates from human faecal samples, only 30% of the proteins were of human origin (Verberkmoes et al., 2009). These findings point out how important it is, which method we choose.

Protein extraction is the first step towards proteomics or metaproteomics. Proteomics makes comprehensive quantification and identification of proteins possible, while metaproteomics provides us with the possibility of characterisation of expressed proteins within the sample. Both methods require extensive work and computational power, although effective workflows already exist (Gonzalez et al., 2020).

Our attempt to find out more about the changes on the microbiome resulted in a minor success. We could clearly see that the sample preparation (including lyophilisation) already affects the results of this analysis. Furthermore, we could observe a shift towards an increased alpha diversity of bacterial taxa, similar to the donor faeces. On the other hand, due to the small sample number and the high interindividual variation we could not find statistically significant taxonomic differences. We did not carry out any virus or bacterial phage analyses either, although these are rather significant parts of the microbiome. These additional microbiome analyses will be the undertaken in a separate study.

Although we observed changes in the protein levels and CFU counts during storage, -20°C seems to be suitable for a stool bank, as we did not observe any decline in the success rate over time. Our findings question the need of the use of bacteria containing stool samples. Furthermore, if no bacteria are needed for the effect, we might not need -20°C for storage temperature either, which makes maintaining stool banks even more straightforward. We have to note though, that we have not carried out any tests to compare SCFA and protein profiles after storing the samples on different temperatures. Moreover, clinical trials are needed as well to compare the efficacy of bacteria free stool samples (or samples with lessened bacterial count at least) with different protein and SCFA profiles.

Recently published results with SER-109 (an oral microbiome therapeutic containing live purified Firmicutes spores) showed, that the dysbiosis behind CDI can be treated with specific bacterial strains. This provides us with a new, controllable method for curing CDI with a significantly lower risk of transmitting any diseases to the patients than by using FMT. However, FMT still has an advantage in the treatment in other bowel diseases (e.g. colitis ulcerosa). Furthermore, the price of this new product will be a very important question, as seen in the case of fidaxomicin - another highly effective but expensive treatment option for CDI. In many regions of the world, the high cost of otherwise excellent therapeutics limits the accessibility, in contrast, FMT (especially our method) can still be carried out with basic equipment and at significantly lower cost.

The main goal of our study was to determine, whether our workflow can sufficiently and reliably meet the needs of a clinical centre treating CDI. The workflow has been proven efficient in regards of clinical effectiveness as well as of human resource management. The pilot projects highlighted several factors that have to be improved in order to achieve high quality data. The most important may be the acquisition of clinical samples prior to FMT, since CDI patients tend to deliver stool samples of inhomogeneous consistency. Combined with the rather irregular defecation frequency, this makes consequent sample collection very difficult. A multicentre approach might also be highly beneficial. We see large potential in the SCFA and protein profiling, as well as in the microbiome analysis of CDI patients prior and after FMT.

## Data availability statement

The datasets presented in this study can be found in online repositories. The names of the repository/repositories and accession number(s) can be found below: ENA database, PRJEB56164.

## Ethics statement

The studies involving human participants were reviewed and approved by Hungarian Medical Research Council (ETT-TUKEB), Budapest, Hungary. The patients/participants provided their written informed consent to participate in this study. Written informed consent was obtained from the individual(s) for the publication of any potentially identifiable images or data included in this article.

## Author contributions

AV: Corresponding author, sample preparation, capsule making, data analysis. LM: Data analysis (proteins), interpretation of data. AB: Data analysis, interpretation of data (lipids). DS: Acquisition of data, development of the method (FMT). PK: Development of the method (capsules). SP: Development of the method (capsules). PR: Revision of the work. FS: Data analysis (16S amplicon sequencing), interpretation of data, revision of the work. BK: Design and revision of the work. ZP: Design and revision of the work, development of the method (FMT). LM acknowledges the financial support of the PTE-AOK-KA-2019-08 grant. All authors contributed to the article and approved the submitted version.
